# Anti-mitochondrial Tryparedoxin Peroxidase Monoclonal Antibody-Based Immunohistochemistry for Diagnosis of Cutaneous Leishmaniasis

**DOI:** 10.3389/fmicb.2021.790906

**Published:** 2022-02-28

**Authors:** Mariana Lourenço Freire, Felipe Dutra Rego, Karine Ferreira Lopes, Lucélia Antunes Coutinho, Rafaella Fortini Queiroz Grenfell, Daniel Moreira Avelar, Gláucia Cota, Marcelo Antônio Pascoal-Xavier, Edward Oliveira

**Affiliations:** ^1^Instituto René Rachou, Fundação Oswaldo Cruz, Belo Horizonte, Brazil; ^2^Faculdade de Medicina, Universidade Federal de Minas Gerais, Belo Horizonte, Brazil

**Keywords:** cutaneous leishmaniasis, immunohistochemistry, diagnosis, monoclonal antibody, mTXNPx

## Abstract

Cutaneous leishmaniasis (CL) remains a globally spreading public health problem. Among Latin America countries, Brazil has the greatest number of recorded CL cases with several *Leishmania* species being associated with human cases. Laboratory diagnosis is one of the major challenges to disease control due to the low accuracy of parasitological techniques, the restricted use of molecular techniques, and the importance of differential diagnosis with regard to several dermatological and systemic diseases. In response, we have developed and validated an immunohistochemistry (IHC) technique for CL diagnosis using anti-mTXNPx monoclonal antibody (mAb). Recombinant *Leishmania*–mTXNPx was produced and used as an immunogen for mAb production through the somatic hybridization technique. The viability of mAb labeling of *Leishmania* amastigotes was tested by IHC performed with skin biopsies from hamsters experimentally infected with *Leishmania amazonensis*, *Leishmania braziliensis*, and *Leishmania guyanensis*. The enzymes horseradish peroxidase (IHC-HRP) and alkaline phosphatase (IHC-AP), both biotin-free polymer detection systems, were used in the standardization step. The IHC was further validated with skin biopsies from 49 CL patients diagnosed by clinical examination and quantitative real-time polymerase chain reaction and from 37 patients presenting other dermatological infectious diseases. Other parasitological techniques, such as direct examination and culture, were also performed for confirmed CL patients. Histopathology and IHC were performed for all included patients. Overall, the highest sensitivity was observed for IHC-AP (85.7%), followed by IHC-HRP (79.6%), direct examination (77.6%), histopathological examination (HE; 65.3%), and *in vitro* culture (49%). Only IHC and HE presented specificity over 90% and were able to detect CL patients regardless of parasite burden (odds ratio > 1.94; 95%CI: 0.34–11.23). A significant increase in positivity rates was observed when IHC-AP was combined with direct examination (95.9%) and HE (93.9%). The IHC techniques evaluated in here detected the main *Leishmania* species causing CL in Brazil and can support diagnostic strategies for controlling this neglected disease, especially if used in combination with other approaches for an integrative laboratorial diagnosis.

## Introduction

Cutaneous leishmaniasis (CL) is a neglected global disease that is often prevalent in the poorest and marginalized communities. It causes skin lesions, residual scars, and stigmatization, with serious psychosocial impacts on the lives of patients (Karimkhani et al., 2016; Galvão et al., 2019). The disease is endemic in at least 88 countries, and more than 210,000 cases have been reported in Brazil during the last decade, mainly caused by *Leishmania* (*Viannia*) *braziliensis*, *Leishmania* (*Leishmania*) *amazonensis*, and *Leishmania* (*Viannia*) *guyanensis* (Brasil, 2017, 2021; WHO, 2020). Considering the clinical complexity of CL and the ineffective strategies available for vector control, disease prevention still relies on early diagnosis, followed by prompt and effective treatment of human cases (PAHO, 2019).

The clinical diagnosis of CL, although relevant, is insufficient for case definition, and differential diagnosis is required due to the broad clinical spectrum of the disease and the often reported presence of similar dermatological diseases in leishmaniasis endemic areas (Tirelli et al., 2017). Laboratory diagnosis is currently based on parasitological, molecular, histopathological, and immunopathological tests; however, gold-standard tests are not yet available (Faber et al., 2003; Goto and Lindoso, 2010). Direct examination of skin lesion scrapings or impression smears is conventionally used as a diagnostic test, even with its variable and generally low sensitivity. In New World countries, where CL chronic cases are frequent, the sensitivity of this test has ranged 30–80%, varying according to the onset of skin lesion, parasite burden, and professional expertise (Ramirez et al., 2000; Schubach et al., 2001; Faber et al., 2003; de Mello et al., 2011; Espir et al., 2016). Polymerase chain reaction (PCR) is usually more sensitive than parasitological tests and allows the identification and quantification of the parasite in tissue. However, despite several advances, the high cost and absence of a standardized protocol limit the use of PCR at reference centers (Moreira et al., 2018). The Montenegro Skin Test has long been used in Brazil as a screening method in endemic areas and in the laboratory routine for CL diagnosis, but the test is no longer used due to the suspension of antigen production (Braz, 2019). Although immunological methods are not currently used in clinical practice, different antigens have been evaluated to improve the restricted scenario for CL diagnosis (Freire et al., 2021), including peroxidoxin (Menezes-Souza et al., 2014), renamed as mitochondrial tryparedoxin peroxidase (mTXNPx) in trypanosomatids (Teixeira et al., 2015). This member of an antioxidant protein family from *Leishmania* is highly expressed in amastigote forms and has been detected in the immunochromatographic assay CL Detect™ Rapid Test (InBios International Inc., Seattle, WA, United States), with sensitivity of around 65% in Old World countries (Bennis et al., 2018; Vink et al., 2018).

Histopathological examination (HE), a widely available technique, is usually more affordable than other assays and can help with CL diagnosis. However, recognizing the amastigote forms of *Leishmania* can occasionally be a limiting factor for CL case confirmation. From this perspective, immunohistochemistry (IHC) has proven to be a valuable tool at reducing this lacuna in CL diagnosis by labeling the amastigote forms of *Leishmania* spp., with sensitivity ranging 60–80% worldwide. Although several advances have been reported using IHC, hyperimmune sera and detection systems based on biotin are still used for CL diagnosis, which may be related to unspecific markings and limited specificity (Salinas et al., 1989; Schubach et al., 2001; Ramos-Vara et al., 2008; Amato et al., 2009; Quintella et al., 2009; Lunedo et al., 2012; Alves et al., 2013; Marques et al., 2017; Gonzalez et al., 2019). Due to the shortage of commercially available monoclonal antibody (mAb) for *Leishmania* detection, the use of IHC is indeed still limited, although promising (Beena et al., 2003; Salotra et al., 2003; Shirian et al., 2014). Thus, we produced an anti-mTXNPx mAb and applied it in the IHC using two biotin-free polymer detection systems for CL diagnosis. The availability of this diagnostic tool represents a potential advance toward increasing access to adequate laboratory diagnosis in Brazil.

## Materials and Methods

### Ethics Statement

The study was approved by the Human Research Ethics Committee of the Instituto René Rachou, Oswaldo Cruz Foundation (IRR/Fiocruz, CAAE number 56188716.5.0000.5091) and of the Universidade Federal de Minas Gerais (UFMG, CAAE number 14887414.0.0000.5149), respectively.

Furthermore, BALB/c mice (*Mus musculus*) and golden hamsters (*Mesocricetus auratus*) were obtained from the IRR/Fiocruz animal facility, and all protocols were licensed by the Ethics Committee of Animal Use of Fiocruz (licenses LW-15/15 and LW-4/18).

### Experimental Design

Since mTXNPx has been considered a promising target in different immunological tests for CL diagnosis (Freire et al., 2021), the recombinant protein *Leishmania* mTXNPx was produced and used to immunize BALB/c mice, stimulating the production of a specific antibody. After this stage, the anti-mTXNPx mAb was produced and used in IHC to label *Leishmania* spp. amastigotes. A pilot experimental study performed IHC with skin biopsies of hamsters experimentally infected with the main species of *Leishmania* associated with CL in Brazil: *L. braziliensis*, *L. guyanensis*, and *L. amazonensis*. Amastigote labeling was evaluated using two IHC protocols based on distinct polymer detection systems. Once the specific labeling was confirmed, these protocols were standardized and validated on skin biopsies from patients with CL-suggestive clinical diagnosis: the first using the enzyme horseradish peroxidase (HRP) and the other using alkaline phosphatase (AP).

### Production of Recombinant Antigen

The nucleotide sequences that code for *Leishmania infantum*–mTXNPx were obtained by back-translation of amino acid sequences (PDB ID: 6E0F). This sequence is highly similar to mTXNPx from other *Leishmania* species associated with CL worldwide, such as *L. braziliensis* (86.7%), *L. amazonensis* (98.7%), *L. guyanensis* (86.3%), and *L. major* (98.2%), such that some are still being named peroxidoxin (Supplementary Figure 1). The restriction sites for *Bam*HI and *Hin*dIII were inserted into the 5′ and 3′ ends of the minigene, respectively. Optimization, synthesis, and insertion of the minigene within the pET28a vector were performed by GenScript (Piscataway, NJ, United States). This synthetic gene was constructed containing the DNA sequence of 678 bp, corresponding to 226 amino acids (24.86 kDa). The pET28a with mTXNPx sequence construction (pET28a-mTXNPx) was used to transform *Escherichia coli* BL21 Star competent cells by thermal shock (15 min on ice, 1 min at 42°C, and 10 min on ice). Thereafter, the selected clones were tested by restriction analysis with *Bam*HI and *Hin*dIII, and those presenting the mTXNPx gene were cultured *in vitro* at 37°C in Luria–Bertani (LB) medium containing 30 μg/ml of kanamycin Sigma-Aldrich, (San Luis, MO, United Estates).

The started cultures were inoculated overnight in fresh LB medium (proportion of 1:25), maintained under the conditions previously described, until an optical density from 0.6 to 0.8 at 590 nm (OD_590_) for the expression of mTXNPx. At this time, the cultures were induced with 1 mmol/L isopropyl-β-D-thiogalactopyranoside (IPTG) under constant agitation of 200 × *g* per min at 37°C overnight. Cells were harvested by centrifugation at 10,000 × *g* for 20 min, and the pellets were lysed for protein purification from inclusion bodies under denaturing conditions (Ntumngia and Adams, 2012). Briefly, the cells were resuspended in lysis buffer [50 mmol/L Tris buffer, pH 8.0, 500 mmol/L NaCl, 0.2 mmol/L EDTA, 3% sucrose, 1% Triton X-100, 200 μg/ml lysozyme, 1 mmol/L phenylmethylsulfonyl fluoride (PMSF), and 20 μg/ml DNAse], and the lysates were sonicated in an ultrasonic processor (VC-750, SONICS VIBRA CELL, Newtown, OH, Unites States) by five cycles of 30-s pulse at 40% of intensity. Inclusion bodies were recovered by centrifugation at 13,000 × *g* for 40 min, washed twice [50 mmol/L Tris buffer (pH 8.0), 3 mol/L urea, 0.2 mmol/L EDTA, and 500 mmol/L NaCl], sonicated, and centrifuged as previously described. The inclusion bodies were solubilized (10 mmol/L phosphate buffer, pH 7.8, 200 mmol/L NaCl, 10 mmol/L Tris, 6 mol/L GuHCl, and 10 mmol/L BetOH), and the recombinant proteins were purified by affinity chromatography using Ni Sepharose High Performance resin (GE Healthcare Life Sciences, Uppsala, Sweden) in Poly-Prep Chromatography columns (Bio-Rad Laboratories, Richmond, CA, United States), according to the instructions of the manufacturer.

The expression and purification of mTXNPx were analyzed by 15% SDS-PAGE (Laemmli, 1970). The protein was blotted to a nitrocellulose membrane (Amersham Protran, 0.45 μm—GE Healthcare, Little Chalfont, United Kingdom), and Western blotting analysis (Towbin et al., 1979) was performed using monoclonal 6x-His-tag antibody (1:3,000) Thermo Fisher Scientific, (Waltham, MA, United Estates) and ECL™ Prime Western Blotting (GE Healthcare UK Ltd., Buckinghamshire, United Kingdom) as a detection agent. Blot images were obtained with ImageQuant LAS 4000 imaging system (GE Healthcare, Chicago, IL, United States).

### Production of Monoclonal Antibody

Two female 5-week-old BALB/c mice were subcutaneously immunized with the first dose containing 20 μg of mTXNPx and Freund’s complete adjuvant. Four other immunizations containing 20 μg of mTXNPx and Freund’s incomplete adjuvant were performed at intervals of 2 weeks. As a control, two female BALB/c mice were also immunized with saline and Freund’s adjuvant, following the same conditions. Blood samples were obtained before each immunization, by submandibular vein puncture using a sterile single-use lancet, as well as after the fifth dose. The serum was titrated by indirect enzyme-linked immunosorbent assay (ELISA), using mTXNPx as antigen and following the protocol described by Grenfell et al. (2014), with changes in absorbance being monitored at a wavelength of 450 nm. Once the production of specific antibodies was confirmed by ELISA, additional boosting immunization was performed intraperitoneally following the conditions described previously. Splenocytes were isolated for cell fusion 3 days after boosting, following the protocols described previously with modifications (Kennett et al., 1979; Harn et al., 1984). Briefly, 3.5 × 10^7^ splenocytes and 7 × 10^6^ Sp2/0-IL6 myeloma cells were mixed with 20 ml Dulbecco’s Modified Eagle Medium (DMEM; Sigma-Aldrich, St. Louis, MO, United States) and centrifuged at 10,000 × *g* for 20 min, discarding the supernatant. After this step, a polyethylene glycol–dimethyl sulfoxide solution (Hybri-Max, Sigma, St. Louis, MO, United States) was preheated to 37°C, and 700 μl was added dropwise into the cell pellet while mixing slowly. The cellular suspension was incubated for 1.5 min, then 15 ml of DMEM was further added, and additional centrifugation at 300 × *g* for 3 min was performed. The cells were resuspended in 30 ml of DMEM, supplemented with 20% fetal bovine serum (FBS) and 1% PenStrep (Gibco Laboratories, Waltham, MA, United States). The fused cells were cultured at 37°C with 5% CO_2_ for 24 h. Selective 2% hypoxanthine–aminopterin–thymidine medium (HAT; Sigma-Aldrich) was added to the fused cell cultures, which were subsequently seeded into a 96-well plate containing previously collected mouse peritoneal macrophages. The wells were first screened by microscopy at 10–14 days later, and antibody production was confirmed by ELISA. The well containing the greatest number of cells and the higher value of absorbance (at least 1.20) was expanded and subcloned by limiting the dilution until at the concentration of a single cell per well. The clone was expanded *in vitro* under the same conditions of the culture, and the supernatant containing anti-mTXNPx mAb was collected weekly. The cell culture supernatant, containing mAb, was concentrated using Amicon^®^ Stirred Cells filtration system with Diaflo ultrafilters (EMD Millipore Corporation, Billerica, MA, United States). The mAb was purified using HiTrap Blue HP and HiTrap rProtein A FF (GE Healthcare, Uppsala, Sweden) according to the instructions of the manufacturer. The purification of anti-mTXNPx mAb was analyzed by 15% SDS-PAGE, and the antigen–antibody reaction was confirmed by Western blotting analysis using 1 μg of mTXNPx, 1:100 of anti-mTXNPx mAb, and 1:20.000 of anti-mouse antibody conjugated with HRP.

### Soluble *Leishmania* Antigen

Soluble *Leishmania* antigens (SLAs) were prepared with stationary-phase promastigotes of *L. amazonensis* (SLaA) (IFLA/BR/67/PH8), *L. braziliensis* (SLbA) (MHOM/BR/75/M2903), and *L. guyanensis* (SLgA) (MHOM/BR/75/M4147) maintained in the biphasic medium NNN (Novy, McNeal, and Nicolle)/LIT (liver infusion tryptose), supplemented with 20% inactivated FBS and 1% PenStrep. The parasites were washed twice with sterile phosphate-buffered saline (PBS) and centrifuged for 10 min at 2,000 × *g* and 4°C, and the supernatants were removed. Pellets containing the parasites were resuspended in lysis buffer (20 mmol/L HEPES, 10 mmol/L KCl, 1.5 mmol/L MgCl_2_, 250 mmol/L sucrose, 1 mmol/L DTT, and 0.1 mmol/L PMSF) and submitted to 10 cycles of liquid nitrogen freezing and heating at 37°C. After this step, the partially lysed cells were centrifuged for 10 min at 2,000 × *g* and 4°C, the supernatant was removed, and the pellets were sonicated in five cycles of 30 s per pulse at 40% intensity, followed by a final centrifugation at 2,000 × *g* and 4°C for 15 min. The capacity of anti-mTXNPx mAb to detect this antigen in SLAs from each *Leishmania* species was performed by Western blotting analysis using 5 μg of each SLA, 1:100 of anti-mTXNPx mAb, and 1:20.000 of anti-mouse antibody conjugated with HRP.

### Pilot Experimental Study Using Golden Hamsters

Male golden hamsters (*Mesocricetus auratus*), 5 weeks old, were experimentally infected with *L. amazonensis* (IFLA/BR/67/PH8), *L. braziliensis* (MHOM/BR/75/M2903), and *L. guyanensis* (MHOM/BR/75/M4147). All inoculums were prepared in a total volume of 200 μl of sterile PBS with 1 × 10^6^ stationary-phase parasites administered intradermally in the dorsal region of the right hind paw (Rêgo et al., 2018). Fragments from skin lesions were harvested at 30 days post-infection and fixed in 10% buffered formalin (pH 7.2). These samples were used in IHC with anti-mTXNPx mAb and two biotin-free polymeric detection systems to confirm *Leishmania* amastigote labeling.

### Validation in Human Samples

#### Study Population

The sample size was calculated considering 57.8% of *Leishmania* prevalence in the Leishmaniasis Reference Center of the Instituto René Rachou, Oswaldo Cruz Foundation (CRL-IRR/Fiocruz), with 0.75 confidence limit and 95% specificity. The minimum values of 34 non-cases and 47 CL cases were estimated (Flahault et al., 2005).

All biopsy samples were from patients with suspected CL and who reside in Minas Gerais, a Brazilian state endemic for this disease. Forty-nine patients who attended from 2017 to 2019 at the CRL-IRR/Fiocruz were included. The CL case definition criterion was based on the detection of the kDNA minicircle of *Leishmania* spp. by quantitative real-time polymerase chain reaction (qPCR). Moreover, direct examination and *in vitro* culture for *Leishmania* spp. isolation were performed by CRL-IRR/Fiocruz for all CL cases. Thirty-seven patients who were attended at the Clínica de Dermatologia Osvaldo Costa (Hospital das Clínicas/Universidade Federal de Minas Gerais), presenting other dermatological infectious diseases clinically similar to CL in the clinical practice, such as sporotrichosis (*n* = 7), paracoccidioidomycosis (*n* = 1), mycobacteriosis (*n* = 6), and dermatitis (*n* = 23), were included as a non-CL group. The definitive diagnosis of these diseases was established by clinical examination and laboratory tests, such as culture for sporotrichosis, paracoccidioidomycosis, and mycobacteriosis as well as search of acid–alcohol-resistant bacilli in dermal smears for mycobacteria cases. For all suggestive clinical examination and laboratory tests with proven negative results, the diagnosis of non-specific dermatitis was determined.

Histopathology and IHC were performed for all included patients. All samples were anonymized, and the researchers involved in the study were blinded to the nature of the samples.

#### Histopathology

Skin fragments from infected hamsters and CL patients were fixed in 10% buffered formalin (pH 7.2) for at least 24 h, followed by histological processing (dehydration, clarification, and embedding in paraffin) using the tissue processor PT05 TS Automatic Tissue Processor and the Inclusion Center CI 2014 (Lupetec, São Carlos, SP, Brazil). Four-micron-thick sections from the paraffin blocks containing the tissues were stained with hematoxylin–eosin and examined with a Zeiss microscope (Hallbergmoos, Germany) to detect the presence of amastigote forms (Pascoe and Gatehouse, 1986).

#### Immunohistochemistry

Since the detection systems can influence the sensitivity of the technique (Skaland et al., 2010), the IHC was standardized and validated with two biotin-free polymeric detection systems: (1) Novolink Polymer Detection System (Leica Microsystems, Newcastle, United Kingdom), a polymeric HRP-linker antibody conjugate system containing 3.3′- diaminobenzidine (DAB) solution as substrate chromogen, which labels the detected antigen with a brown color—IHC using this detection system is hereon referred to as IHC-HRP and (2) Bond Polymer Refine Red Detection (Leica Microsystems, Newcastle, United Kingdom), a polymeric AP-linker antibody conjugate system containing Fast Red solution as substrate chromogen, which labels the antigen-detected in red color—IHC using this detection system is hereon referred to as IHC-AP.

For all IHC, 4-μm-thick sections of the paraffin blocks containing the tissues were mounted on ImmunoSlides (EasyPath, São Paulo, SP - Brazil) and incubated overnight at 56°C. Initially, the IHC reactions (IHC-HRP and IHC-AP) were standardized using fragments of skin lesions from hamsters infected with *L. amazonensis*, *L. braziliensis*, and *L. guyanensis* and from three CL patients previously confirmed by direct parasitological examination, *Leishmania* culture, and histopathology. Relevant parameters of the reactions, such as antigen retrieval, endogenous peroxidase blocking, antibody dilution, and incubation with the substrate chromogen, were evaluated to obtain a strong staining signal, with minimal or no background staining, using the higher antibody dilution in shorter reaction time (Lin and Chen, 2014). Details about the standardization are presented in Supplementary Table 1. The final protocols for IHC with each polymeric detection system are described below.

##### Immunohistochemistry-Horseradish Peroxidase

Microscope slides were dewaxed and hydrated with two xylene washes (10 min at room temperature), three 100% ethanol washes, and two distilled water washes (5 min for each step). The reaction proceeded with the following steps: (1) antigenic recovery with 10 mmol/L citrate buffer solution, pH 6.0, at a temperature of approximately 90°C for 30 min using a steamer, (2) inhibition of the endogenous peroxidase with peroxidase block for 20 min, (3) blocking of non-specific sites with protein block for 40 min, (4) incubation with anti-mTXNPx mAb (1:300 dilution) for 60 min, (5) post-primary antibody block for 30 min, (6) Novolink Polymer incubation for 30 min, (7) reaction revelation with DAB Chromogen (1:100 dilution in Novolink DAB substrate buffer) for 30 s, and (8) counterstaining with hematoxylin for 3 min.

##### Immunohistochemistry-Alkaline Phosphatase

Dewaxing, hydrating, and antigenic recovery were performed using 1:100 dilution of Trilogy^®^ (Cell Marque, Rocklin, CA, United States) at a temperature of approximately 90°C for 30 min using a steamer. The reaction proceeded with the following steps: (1) blocking of non-specific sites with 5% powdered and skimmed milk (Molico^®^, Nestle) in PBS for 30 min, (2) incubation with anti-mTXNPx mAb (1:500 dilution) for 60 min, (3) post-primary AP antibody block for 30 min, (4) polymer AP incubation for 30 min, (5) reaction revelation with a solution containing Red Part A (1:10), Red Part B (1:50), and Red Part C (1:50), diluted in Red Part D for 3 min, and (6) counterstaining with hematoxylin for 3 min.

All steps of IHC-HRP and IHC-AP were performed at room temperature after the antigenic recovery, and Tris-buffer (Tris base 5 mmol/L; NaCl 140 mmol/L; pH 7.6) was used as washing solution between each step. The slides were cover-slipped with Entellan^®^ (Merck) and examined with a Zeiss microscope (Hallbergmoos, Germany) to detect the presence of amastigote forms (Pascoe and Gatehouse, 1986).

### Quantitative Real- Time Polymerase Chain Reaction

Whole DNA was extracted from skin biopsies of patient and from *L. braziliensis* promastigote culture using Wizard^®^ SV Genomic DNA Purification System according to the instructions of the manufacturer Promega, (Madison, WI, United Estates). A 120-bp fragment from a variable kDNA region of the *Leishmania* genus was amplified using the primers 150 (sense) 5′ (C/G)(C/G)(G/C) CC(C/A) CTA T(T/A)T TAC ACC AAC CCC 3′ and 152 (antisense) 5′ GGG GAG GGG CGT TCT GCG AA 3′ (Degrave et al., 1994; Passos et al., 1996). Amplifications were performed in triplicate containing 12.5 μl Universal SYBR Green PCR master mix (Applied Biosystems, Foster City, CA, United States), 1 mmol/L of each primer (Integrated DNA Technologies, Coralville, IA, United States), and 10 ng of DNA template for a final volume of 25 μl. Real-time PCR was performed with the Step One Real-Time PCR System (Applied Biosystems, Foster City, CA, United States), using optical grade 96-well plates. After an initial denaturation step of 10 min at 95°C, 30 cycles of 15 s at 95°C, 1 min at 60°C, and 30 s at 72°C, the melt curve was determined (Chagas et al., 2021). PCR mix without DNA and DNA extraction kit components were used in all qPCR assays as negative controls. Standard curves were obtained using serial dilutions (1:10 dilution factor) of *L. braziliensis* (MHOM/BR/75/M2903), starting from the concentration equivalent to 1 ng until a concentration equivalent to 1 × 10^–6^ ng. The parasite burdens of skin biopsies were calculated considering 83.15 fg of *Leishmania* DNA equivalent to one parasite due to the size of the *L. braziliensis* haploid genome, following the calculation (parasite DNA equivalents per reaction/amount of tissue DNA per reaction) × 10^3^, expressed as *Leishmania* parasites per microgram of tissue DNA (Jara et al., 2013; Suárez et al., 2015; Sevilha-Santos et al., 2019).

### Data Analysis

Sensitivity, specificity, and diagnostic accuracy were calculated using a two-by-two contingency table with an exact 95% binomial confidence interval (CI) using Open-Epi Software (Dean et al., 2015). These parameters were compared using the chi-square (χ^2^) test at the confidence level of 0.05, with MedCalc for Windows, version 15.0 (MedCalc Software, Ostend, Belgium).

Binary logistic regression analysis was performed to evaluate the influence model of the following predictors (variables) in the positivity of the tests: gender and age of the patients as well as the site, number, and onset time of skin lesions and parasite burden. Significant predictors were selected by the forward stepwise method, considering *P*-value < 0.25, from an initial modeling considering all these variables, and odds ratios (OR) with 95% confidence intervals were calculated. All analyses were performed in Minitab 17 (State College, PA, United States).

## Results

### Characteristics of Participants

Forty-nine samples from CL patients with clinically compatible lesion and positive qPCR were included (Supplementary Table 2). The mean age was 44.8 years (SD ± 20.6), 75.5% (37/49) of the patients were male, and the mean number of lesions per patient was 2 (SD ± 2.18). The most predominant lesion sites were the lower extremities (61.9%), followed by the upper extremities (23.8%) and the face (9.5%). The average lesion onset time was 4 months (SD ± 3.04), in which patients who presented ≤2 months were considered with recent infection (*n* = 16) and patients who presented >2 months were defined with longer evolution (*n* = 33), according to the cytokine profile during the early phase of the disease (Ribeiro-de-Jesus et al., 1998; Baratta-Masini et al., 2007).

### Expression and Purification of mTXNPx

The fragment of 226aa corresponding to *L. infantum*–mTXNPx was used to obtain the DNA sequence by reverse translation, which was optimized for expression in prokaryotic systems (Supplementary Figure 2). The transformation of *E. coli* with the synthetic gene of approximately 678 bp was confirmed by enzymatic cleavage. Protein expression was confirmed through the difference between the bacterial lysate before and after IPTG induction in 15% SDS-PAGE, and mTXNPx purification was confirmed by reactivity with 6x-His-tag antibody (Supplementary Figure 3).

### Production of Monoclonal Antibody

**FIGURE 1:**
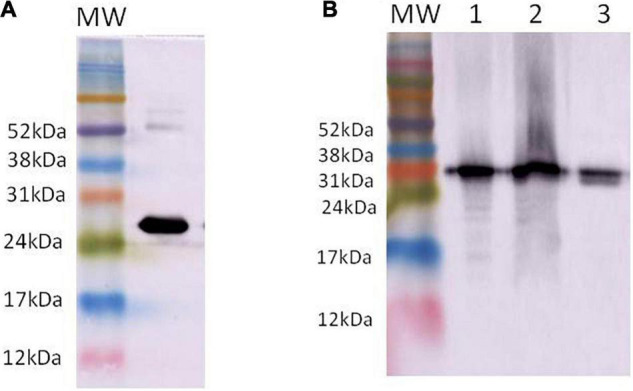
Western blotting presenting the recognition of recombinant mTXNPx **(A)** and native mTXNPx **(B)** present in the soluble antigen from *L. amazonensis* (1), *L. braziliensis* (2), and *L. guyanensis* (3) by anti-mTXNPx monoclonal antibody. Molecular weight (MW) markers: Amersham ECL Rainbow Markers (GE Healthcare, Chicago, IL, United States).

A significant production of anti-mTXNPx antibodies was observed in mice before the third immunization, and the absorbance remained greater than 1.0 after five immunizations. About 400 HAT-resistant hybridoma clones were recovered after cell fusion between BALB/c mice splenocytes and Sp2/0-IL6 myeloma cells. Of this total, 21 produced a high-binding mTXNPx antibody. The anti-mTXNPx mAb was purified and confirmed by 15% SDS-PAGE (Supplementary Figure 4). The specificity of the anti-mTXNPx mAb to recognize mTXNPx was analyzed (Figure 1A) as well as the presence of this native antigen in SLaA, SLbA, and SLgA (Figure 1B).

### Pilot Experimental Study

**FIGURE 2:**
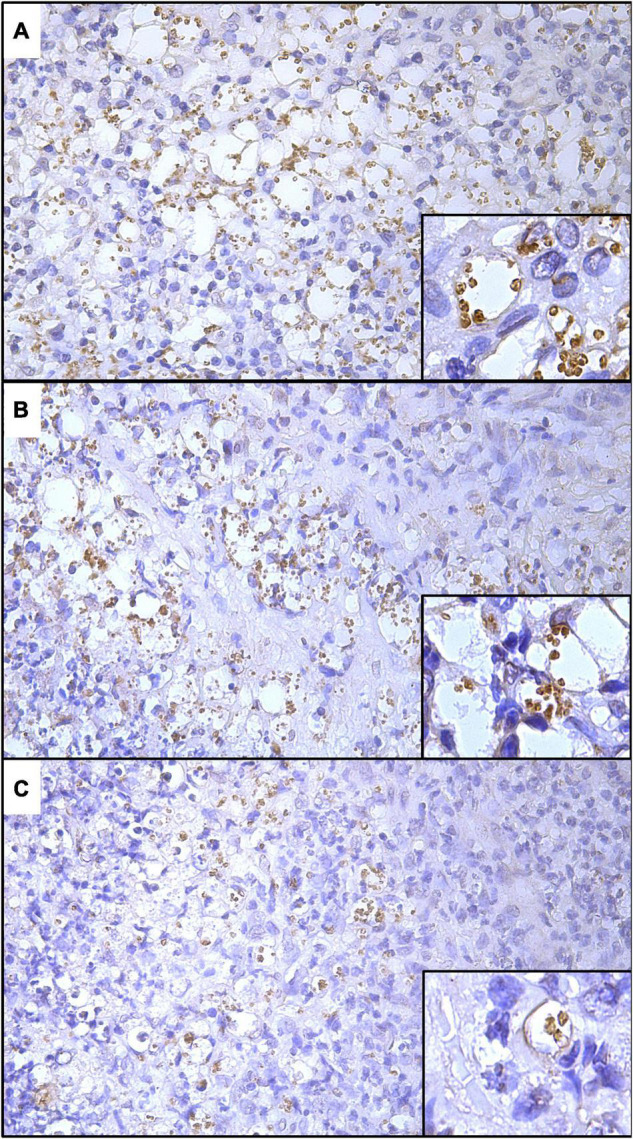
Histological sections of skin lesions from golden hamsters experimentally infected with *L. amazonensis* **(A)**, *L. braziliensis* **(B)**, and *L. guyanensis* **(C)** showing the labeling of amastigote forms by IHC-HRP using anti-mTXNPx mAb (IHC-HRP, 280×). Detail showing amastigote within parasitophorous vacuoles (700×).

**FIGURE 3:**
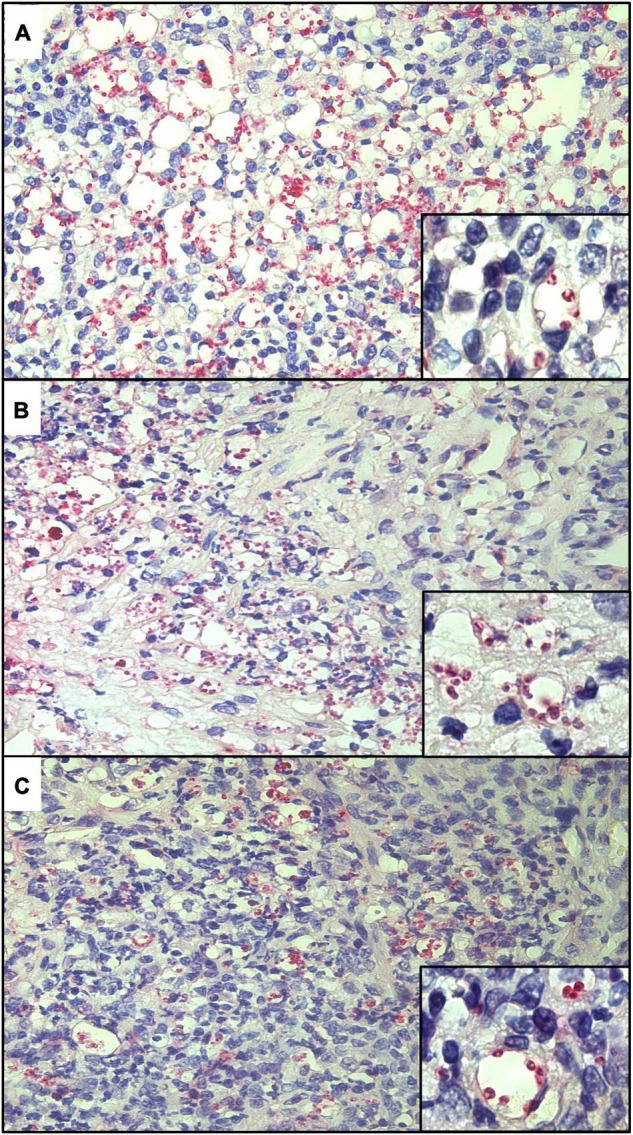
Histological sections of skin lesions from golden hamsters experimentally infected with *L. amazonensis* **(A)**, *L. braziliensis* **(B)**, and *L. guyanensis* **(C)** showing the labeling of amastigote forms by IHC-AP using anti-mTXNPx mAb (IHC-AP, 280×). Detail showing amastigote within parasitophorous vacuoles (700×).

The presence of *L. amazonensis*, *L. braziliensis*, and *L. guyanensis* amastigotes from skin lesion fragments of experimentally infected hamsters was confirmed by IHC using anti-mTXNPx mAb with both detection systems (Figures 2, 3). A histopathological examination of these fragments also confirmed the presence of amastigote forms.

### Performance of the Diagnostic Tests

The sensitivity, specificity, and accuracy of the diagnostic tests are shown in Table 1. The IHC-AP was the only test with sensitivity over 80%, while a significantly lower sensitivity was observed for HE (*p* = 0.02) and culture (*p* = 0.001). No difference was observed between HE and IHC (for both detection systems) for specificity. Overall, the IHC techniques exhibited the highest accuracies, but only IHC-AP had significantly superior results compared to HE (*p* = 0.02). There was no significant difference between IHC-HRP and IHC-AP for any of the evaluated parameters.

**TABLE 1:** Sensitivity, specificity, and diagnostic accuracy of direct examination, culture, histopathology (HE), and immunohistochemistry (IHC) using two detection systems.

Tests	Sensitivity (%) [95% CI] (*n* = 49)	Specificity (%) [95% CI] (*n* = 37)	Accuracy (%) [95% CI] (*n* = 86)
Direct examination^a^	77.6 [64.1–87.0] (38/49)	–	–
Culture^a^	49.0 [35.6–62.5] (24/49)	–	–
HE	65.3 [51.3–77.1] (32/49)	94.6 [82.3–98.5] (35/37)	77.9 [68.1–85.4]
IHC-HRP	79.6 [66.4–88.5] (39/49)	94.6 [82.3–98.5] (35/37)	86.1 [77.2–91.8]
IHC-AP	85.7 [73.3–92.9] (42/49)	97.3 [86.2–99.6] (36/37)	90.7 [82.7–95.2]

*^a^Direct examination and culture were not performed for the non-CL group; therefore, the specificity and accuracy of those were not calculated.*

**FIGURE 4:**
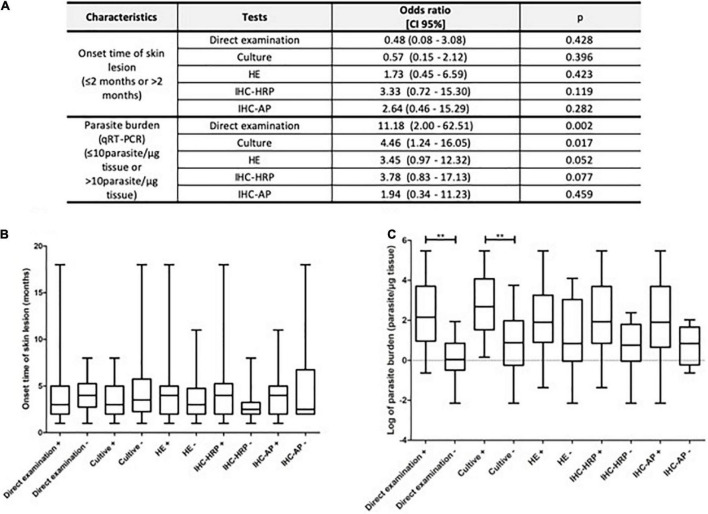
Relationship between test positivity and skin lesion onset time and parasite burden. Multivariate analyses: **(A)** graphic demonstration of skin lesion onset time **(B)** and parasite burden **(C)** according to the results of the diagnostic tests (**P ≤ 0.001).

Skin lesion onset time (≤ or >2 months) and parasite burden (≤ or >10 parasites/μg tissue) were the only parameters correlated with diagnostic test assertiveness (Figure 4A). Although all tests were affected by the onset time of lesion, this parameter was not a determinant of assertiveness (Figure 4B). In contrast, high parasite burden (≥10 parasites/μg of tissue) was significantly associated with positivity rate only for direct examination (OR = 11.18; 95%CI = 2.00–62.51) and culture (OR = 4.46; 95%CI = 1.24–16.05). However, parasite burden was not observed to affect the assertiveness of the diagnosis for HE and IHC, suggesting that these tests were not affected by this parameter. Significant differences between parasite burden and the results of direct examination and culture are also shown in Figure 4C.

**FIGURE 5:**
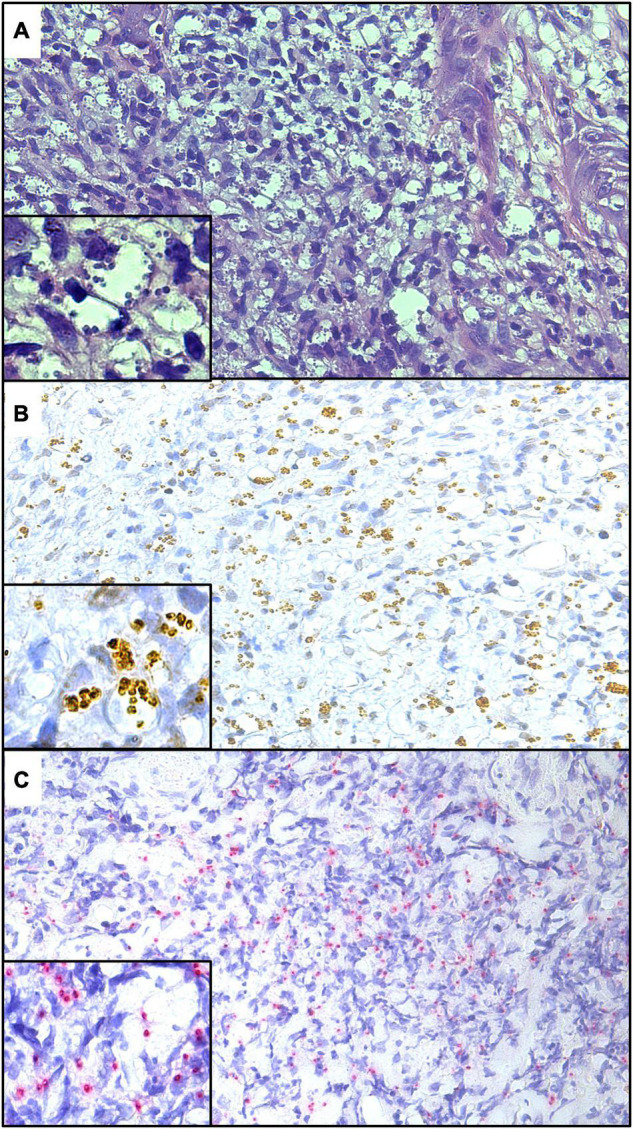
Histological sections of skin lesions from patients with high parasite burden, stained by hematoxylin–eosin **(A)**, IHC-HRP **(B)**, and IHC-AP **(C)** (280×). Detail showing amastigote within parasitophorous vacuoles (700×).

**FIGURE 6:**
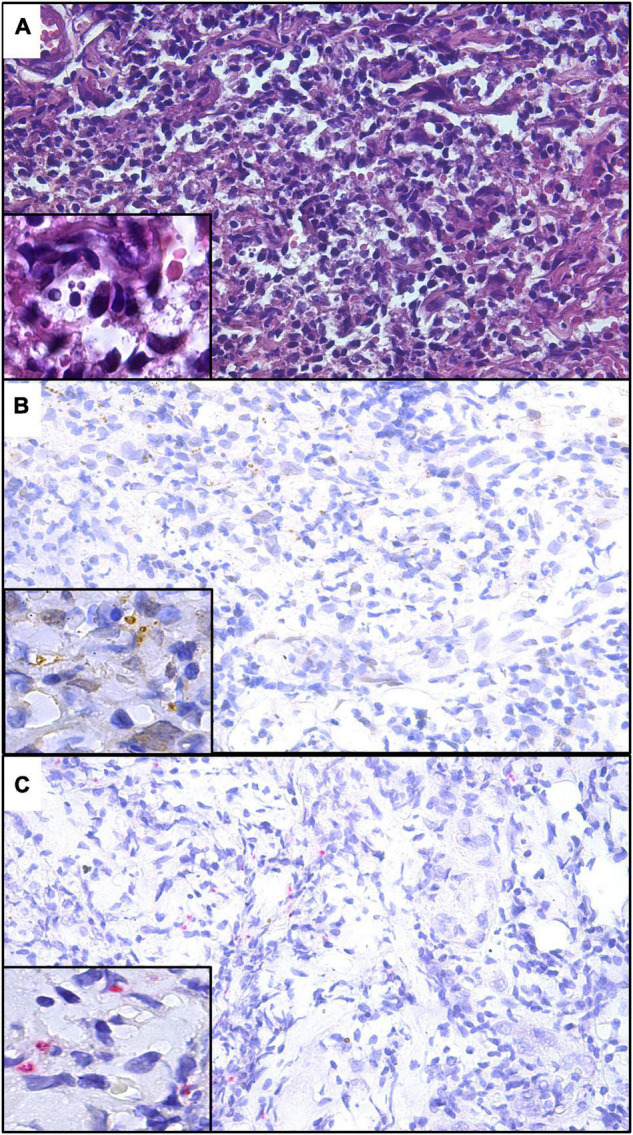
Histological sections of skin lesions from patients with low parasite burden, stained by hematoxylin–eosin **(A)**, IHC-HRP **(B)**, and IHC-AP **(C)** (280×). Detail showing amastigote within parasitophorous vacuoles (700×).

**FIGURE 7:**
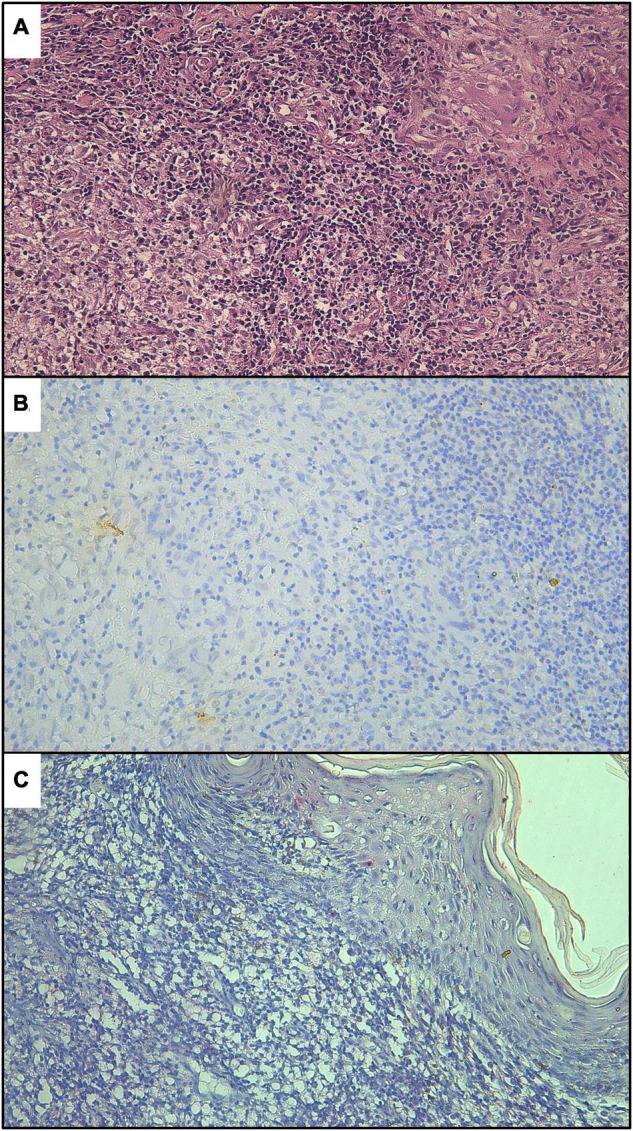
Histological sections of skin lesions from non-cutaneous leishmaniasis patients, stained by hematoxylin–eosin **(A)**, IHC-HRP **(B)**, and IHC-AP **(C)**.

The great improvement of IHC techniques over traditional hematoxylin–eosin staining of sections is due to the ease by which amastigote forms can be visualized in the histological sections, especially for patients with low parasite burdens (Figures 5, 6). This was true regardless of the detection system (HRP or AP), although the chromatic contrast between labeled amastigotes and tissue is greater for IHC-AP. The absence of stained amastigotes in histological sections for non-CL patients can be checked in Figure 7.

Since diagnostic tests are frequently used in combination to enhance the confirmation of CL cases, the positivity rates for combining tests were calculated (Table 2). The use of IHC techniques in association with another diagnostic test increased the positivity rates regardless of the combination, clearly improving the assertiveness of CL diagnosis. An increase in positivity rate when using direct examination was observed only when it was combined with IHC-AP (95.9%; *p* = 0.0079). The combination between HE and IHC-HRP and between HE and IHC-AP resulted in 85.7% (42/49) and 93.9% (46/49) positivity, respectively (*p* < 0.02). Although the inclusion of other tests in parallel, such as culture and/or direct examination, increased the number of CL patients correctly diagnosed, no statistical difference was observed in relation to the results for HE and IHC-HRP or HE and IHC-AP (*p* > 0.05).

**TABLE 2:** Positivity rates obtained by combining diagnostic test results.

	Combined tests		% positivity rates (*n* = 49)	*P*
Two tests	Direct examination 77.6%	Culture	83.7 (41)	0.4470
		HE	87.8 (43)	0.1842
		IHC-HRP	91.8 (45)	0.0521
		IHC-AP	95.9 (47)	0.0079*
	Culture 49%	HE	85.7 (42)	0.0001*
		IHC-HRP	91.8 (45)	<0.0001*
		IHC-AP	87.8 (43)	<0.0001*
	HE 65.3%	IHC-HRP	85.7 (42)	0.0195*
		IHC-AP	93.9 (46)	0.0005*
Three tests	Direct examination + culture 83.7%	HE	93.9 (46)	0.1112
		IHC-HRP	98.0 (48)	0.0146*
		IHC-AP	98.0 (48)	0.0146*
	Direct examination + HE 87.8%	IHC-HRP	91.8 (45)	0.5151
		IHC-AP	95.9 (47)	0.1449
	Culture + HE 85.7%	IHC-HRP	95.9 (47)	0.0822
		IHC-AP	95.9 (47)	0.0822
Four tests	Direct examination + culture + HE 93.9%	IHC-HRP	98.0 (48)	0.3057
		IHC-AP	98.0 (48)	0.3057

*p < 0.05.

## Discussion

Cutaneous leishmaniasis remains a relevant public health problem, leading to psychosocial problems, stigmatization, exclusion, and distress (WHO, 2020). One of the many challenges that have been addressed to reduce the impact of this disease is early and accurate diagnosis, which is often impaired due to the broad range of clinical manifestations, the absence of a gold-standard test, and the scarce economic resources. Indeed further studies on CL diagnosis are essential to reach adequate laboratory testing for at least 80% of the cases, according to the World Health Organization action plan (PAHO, 2017). The present study developed and validated IHC using anti-mTXNPx mAb and two biotin-free polymeric detection systems for CL diagnosis. The results suggest that this technique may significantly improve the sensitivity of CL diagnosis even in patients presenting low parasite burden. Furthermore, IHC with anti-mTXNPx mAb was found to be able to detect the main *Leishmania* species causing CL in Brazil, and so it can be used in routine diagnostic procedures.

*Leishmania* mTXNPx is a member of an antioxidant protein family and has been considered a promising target (McGonigle et al., 1998), especially due to the highly conserved degree and expression level of the protein (Harder et al., 2006; Jirata et al., 2006) with confirmed immunogenic (Webb et al., 1998; Campos-Neto et al., 2001; Coler et al., 2002) and antigenic properties (Santarém et al., 2005; Menezes-Souza et al., 2014). This protein plays a pivotal role in neutralizing reactive oxygen species produced by macrophages as a defense mechanism to eliminate *Leishmania* amastigote forms (Barr and Gedamu, 2003). Although mTXNPx is present in all stages of the development of the parasite, its expression is increased in the amastigote forms of several *Leishmania* species (Castro et al., 2002; Harder et al., 2006; Cuervo et al., 2009; Coelho et al., 2012). Therefore, mTXNPx has been used in immunodiagnostic studies and as vaccine candidate for leishmaniasis (Webb et al., 1998; Campos-Neto et al., 2001; Santarém et al., 2005; Menezes-Souza et al., 2014; Tabatabaie et al., 2014).

Previous studies have shown controversial results of using recombinant mTXNPx for CL diagnosis. In Brazil, high sensitivity (98.5%) has been reported when mTXNPx was used in ELISA (Menezes-Souza et al., 2014). On the other hand, in Suriname, the immunochromatographic assay CL Detect™ Rapid Test (InBios International Inc., Seattle, WA, United States), based on the *L. major* mTXNPx detection, presented low sensitivity (<37%), below that reported in Asia and Africa (De Silva et al., 2017; Vink et al., 2018; Schallig et al., 2019). In this specific context, the low sensitivity was associated with a lower expression of mTXNPx antigen or even due to a variant of this antigen in *L. guyanensis*, the main species associated with CL in Suriname (Schallig et al., 2019). Here, considering the endemic region, the CL patients evaluated were probably infected with *L. braziliensis*; however, the expression of mTXNPx in promastigote and amastigote forms of other prevalent species in Brazil, such as *L. amazonensis*, *L. braziliensis*, and *L. guyanensis*, was also demonstrated by Western blotting and IHC, respectively. The results suggest that IHC may also be used as a diagnostic test in all CL endemic regions in Brazil or wherever these species occur. It is noteworthy that the mTXNPx sequence used in the present study was obtained from *L. infantum*, and it is possible that the anti-mTXNPx mAb also recognizes amastigotes in patients infected with this parasite or even of other species whose protein sequences are similar, such as *Leishmania donovani*. Although uncommon, these species could also be associated with CL cases (Siriwardana et al., 2013; Lyra et al., 2015).

Several authors have considered IHC as one of the most relevant techniques in CL diagnosis (Salotra et al., 2003; Shirian et al., 2014). However, despite advances, most of the studies to date generally used anti-*Leishmania* serum obtained from immunizing animals with SLA and adopted a detection system using avidin–biotin complex (ABC) or labeled streptavidin–biotin (LSAB) (Salinas et al., 1989; Schubach et al., 2001; Amato et al., 2009; Quintella et al., 2009; Marques et al., 2017; Gonzalez et al., 2019). Notably, polyclonal sera contain different anti-*Leishmania* antibodies, and therefore cross-reactions are commonly reported, reducing the specificity of the reaction, especially in endemic regions. Furthermore, background color is frequently observed due to the presence of endogenous biotin when ABC or LSAB is used (Mighell et al., 2008; Ramos-Vara et al., 2008). For this reason, we propose the use of anti-mTXNPx mAb and polymer detection systems since polymer-based technology is often easier to carry out and presents higher sensitivity and less background staining color compared to biotin detection systems (Ramos-Vara, 2005). We also propose to evaluate distinct polymer detection systems: horseradish peroxidase enzyme, the most widely used, and alkaline phosphatase enzyme, recommended for the diagnosis of infectious diseases (Liang et al., 2007; Ramos-Vara et al., 2008). Although these enzymes presented satisfactory results for CL diagnosis (Table 1) without a significant difference, IHC-AP was found to present better chromatic contrast and, thus, enhance the visualization of amastigote forms. Moreover, it was shown that, in some cases, brown pigments (hemosiderin) within macrophages may cause confusion and confound assertiveness in IHC-HRP as previously reported (Ramos-Vara, 2005).

Quantitative real-time polymerase chain reaction was used as a reference standard test due to its higher sensitivity, and the IHCs presented 79.6% (IHC-HRP) and 85.7% (IHC-AP) sensitivity. However, other studies have reported varying sensitivity, from 58.5 to 80%, for CL diagnosis, considering parasitological tests as a reference standard test and IHC with polyclonal sera and biotin systems (Salinas et al., 1989; Schubach et al., 2001; Amato et al., 2009; Quintella et al., 2009; Marques et al., 2017; Gonzalez et al., 2019). Using histopathological or *in vitro* culture as reference tests, Kenner et al. (1999) reported a sensitivity of 51% for IHC using mAb anti-*Leishmania gerbilli* (G2D10) and ABC system (Kenner et al., 1999). Sensitivity over 90% was reported for IHC using mAb against the specific protein of *L. major* and *Leishmania tropica* in Iran, considering clinical diagnosis as the reference test (Shirian et al., 2014). Regarding the specificity, we evaluated the anti-mTXNPx mAb in samples from patients presenting clinical signs similar to CL, which may represent a confounding factor in the clinical practice. Cross-reactions with other pathogens phylogenetically close to *Leishmania*, causing diseases such as Chagas’ disease, was not evaluated here and may be performed in further studies. Overall, these results suggest that IHC may be a sensitive and effective test for CL diagnosis, especially using specific mAb and signal amplification polymeric systems as proposed here. However, it should be noted that the reference standard test is a crucial parameter in the accuracy of studies as it directly influences the performance of the index test.

The inverse correlation between skin lesion onset time and the sensitivity of parasitological techniques for CL diagnosis has been widely described, and reduction in sensitivity is mainly observed after 6 months of onset time (Weigle et al., 1987; Gutierrez et al., 1991; Ramirez et al., 2000). Multivariate regression analysis showed that the four tests evaluated here were influenced by this parameter; however, this predictor variable was not strictly determinant for the sensitivity of these tests (de Mello et al., 2011). On the other hand, parasite burden, measured by qPCR, was determinant for the positivity of direct examination and *in vitro* culture, highlighting a clear dependence of these tests on parasite burden. Since this parameter was not correlated with the assertiveness of HE and IHCs, their use may be strongly recommended regardless of parasite burden in CL patients.

Here we verify a clear improvement in CL case confirmation when HE was used in combination with IHC as previously reported (Quintella et al., 2009; Gonzalez et al., 2019). Indeed the use of IHC in laboratories with adequate equipment and infrastructure for routine histopathological diagnosis can make parasite identification more feasible. Regarding the histopathological patterns, only 8 (16.32%) samples from CL patients presented a well-organized granulomatous reaction in HE. This low number was not enough for any statistical inferences between the IHC positivity and the organization of the inflammatory response. We also verified in this study that IHC-AP significantly increased the positivity rate of direct examination (from 77.6 to 95.9%); thus, if pathology service is available, the use of direct examination and IHC can provide greater autonomy to the diagnosis procedure (Gonzalez et al., 2019).

In this study, we developed, applied, and evaluated the performance of anti-mTXNPx mAb in IHC for CL diagnosis. High sensitivity and specificity were observed for IHC using HRP and AP polymer detection systems; however, from the perspective of our expertise, we suggest the implementation of IHC-AP as a CL diagnosis tool, especially due to the chromatic contrast for amastigote visualization. Although a significantly substantial performance was presented here, several aspects of IHC using mAb should be considered, such as national production and accessibility. In locations with pathology service, the use of IHC could provide greater autonomy to CL diagnosis.

Thus, we encourage further studies on this topic, especially in endemic areas where other species of *Leishmania* are prevalent, like *L. guyanensis* and *L. amazonensis*. Furthermore, multicenter studies of IHC-HRP and IHC-AP as well as of their performance with mucosal leishmaniasis are recommended. Further analysis considering aspects such as cost-effectiveness is also required as a crucial step to incorporate this technique in health services. Lastly, we believe that this study represents a crucial step toward the broader evaluation required to achieve greater autonomy in CL diagnosis outside of reference centers and decentralizing its diagnosis especially in Brazil.

## Data Availability Statement

All relevant data are within the article/Supplementary Material.

## Ethics Statement

The studies involving human participants were reviewed and approved by Human Research Ethics Committee of the Instituto René Rachou, Oswaldo Cruz Foundation (IRR/Fiocruz, CAAE number 56188716.5.0000.5091). Written informed consent for participation was not required for this study in accordance with the national legislation and the institutional requirements. The animal study was reviewed and approved by Ethics Commission of Animal Use of Fiocruz (licenses LW-15/15 and LW-4/18).

## Author Contributions

MF produced recombinant mTXNPx and monoclonal antibody, performed the qPCR assay, analyzed the results, and wrote manuscript. FR assisted with *Leishmania* cultivation and experimental infection. KL assisted with the recombinant mTXNPx production. LC aided with the monoclonal antibody production. RG supported with the monoclonal antibody production. DA assisted with the qPCR assay and critically reviewed the manuscript for intellectual content. GC aided with the enrollment of the participants and caption of the participant data. MP-X aided validation of immunochemistry technique, supported with the study design, and critically reviewed the manuscript for intellectual content. EO assisted with the study design, data analysis, and critically reviewed the manuscript for intellectual content. All authors contributed to the article and approved the submitted version.

## Conflict of Interest

The authors declare that the research was conducted in the absence of any commercial or financial relationships that could be construed as a potential conflict of interest.

## Publisher’s Note

All claims expressed in this article are solely those of the authors and do not necessarily represent those of their affiliated organizations, or those of the publisher, the editors and the reviewers. Any product that may be evaluated in this article, or claim that may be made by its manufacturer, is not guaranteed or endorsed by the publisher.

## Supplementary Material

Supplementary Figure 1 Sequence of *Leishmania infantum*–mTXNPx used to produce the recombinant antigen, aligned with mTXNPx from *Leishmania braziliensis* **(A)**, *Leishmania amazonensis* **(B)**, *Leishmania guyanensis* **(C)**, and *Leishmania major* **(D)**.

Supplementary Figure 2 Sequences of mTXNPx: **(A)** amino acid sequence of *L. infantum* mTXNPx (PDB ID: 6E0F) containing 226aa. **(B)** Synthetic gene construction containing the DNA sequence (678 bp), restriction enzyme sites *Bam*HI and *Hin*dIII at the 5′ and 3′ ends, respectively, and the stop codon.

Supplementary Figure 3 Production of recombinant mTXNPx. **(A)** Polyacrylamide gel with DNA fragment of synthetic gene stained with silver, **(B)** 15% SDS-PAGE with lysate of culture before (1) and after (2) induction with IPTG and purified recombinant mTXNPx (3). **(C)** Western blotting using monoclonal 6x-His-tag antibody against recombinant mTXNPx. Molecular marker: 100 bp DNA Ladder (Promega, United States); molecular weight markers: Amersham ECL Rainbow Markers (GE Healthcare, Chicago, IL, United States).

Supplementary Figure 4 Production of anti-mTXNPx monoclonal antibody. **(A)** Kinetics of antibody production (pools of sera) measured by ELISA before each immunization from mice immunized with recombinant mTXNPx or saline plus Freund’s adjuvant. **(B)** 15% SDS-PAGE showing purified monoclonal anti-mTXNPx antibody under denaturing conditions (fragments of ∼50 and ∼25 kDa corresponding to heavy and light chains, respectively).
