# Supplementary material for: Anti-mitochondrial Tryparedoxin Peroxidase Monoclonal Antibody-Based Immunohistochemistry for Diagnosis of Cutaneous Leishmaniasis
Source: Front Microbiol. 2022 Feb 28;12:790906. doi: 10.3389/fmicb.2021.790906 (PMC8918995; doi:10.3389/fmicb.2021.790906)
Supplement: Supplementary file 1 [file Table_1.DOCX]

**S1 Table.** Standardization and details on Immunohistochemistry protocols

| **IHC steps** | | **1 Test** | **2 Test** | **3 Test** | **4 Test** | **Final protocol** |
| --- | --- | --- | --- | --- | --- | --- |
| **IHC-HRP¹** | **Fixation** | 56°C overnight | 56°C overnight | 56°C overnight | 56°C overnight | **56°C overnight** |
|  | **Deparaffinization** | Xylene: 2x 20’ | Xylene: 2x 20’ | Xylene: 2x 20’ | Xylene: 2x 20’ | **Xylene: 2x 20’** |
|  | **Rehydration** | Ethanol: 3x 5’ /  Running water 3’ | Ethanol: 3x 5’ /  Running water 3’ | Ethanol: 3x 5’ /  Running water 3’ | Ethanol: 3x 5’ /  Running water 3’ | **Ethanol: 3x 5’ / Running water 3’** |
|  | **Antigen retrieval** | 10mmol/L Sodium citrate pH 6 (30’ in steamer) | Tris/EDTA pH 9 buffer  (30’ in steamer) | 10mmol/L Sodium citrate pH 6 (30’ in steamer) | 10mmol/L Sodium citrate pH 6 (30’ in steamer) | **10mmol/L Sodium citrate pH 6 (30‘ in steamer)** |
|  | **Peroxidase Block** | 10’ | 10’ | 20’ / 40’ | 20’ | **20’** |
|  | **Protein Block** | 10’ | 10’ | 40’ / 60’ | 40’ | **40’** |
|  | **Antibody dilution** | 1:100; 1:300; 1:500; 1:1000 | 1:100; 1:300; 1:500; 1:1000 | 1:300 | 1:300 | **1:300** |
|  | **Post Primary** | 30’ | 30’ | 30’ | 30’ | **30’** |
|  | **Polymer** | 30’ | 30’ | 30’ | 30’ | **30’** |
|  | **Substrate - chromogen** | 50’’ | 50’’ | 50’’ | 15’’; 30’’; 50’’ | **30’’** |
| **IHC-AP²** | **Fixation** | 56°C overnight | 56°C overnight | 56°C overnight | 56°C overnight | **56°C overnight** |
|  | **Deparaffinization** | Trilogy solution³  (20’ in steamer) | Xylene: 2x 20’ | Trilogy solution³  (20’ in steamer) | Trilogy solution³  (20’ in steamer) | **Trilogy solution³**  **(20’ in steamer)** |
|  | **Rehydration** |  | Ethanol: 3x 5’ /  Running water 3’ |  |  |  |
|  | **Antigen retrieval** |  | 10mmol/L Sodium citrate pH 6 (30’ in steamer) |  |  |  |
|  | **Protein block** | 30’ | 30’ | 30’ | 30’ | **30’** |
|  | **Antibody dilution** | 1:100; 1:300; 1:500; 1:1000 | 1:100; 1:300; 1:500; 1:1000 | 1:500 | 1:500 | **1:500** |
|  | **Post Primary** | 30’ | 30’ | 30’ | 30’ | **30’** |
|  | **Polymer** | 30’ | 30’ | 30’ | 30’ | **30’** |
|  | **Substrate-chromogen dilution** | A(1:5); B(1:25); C (1:25) dilution in D for 3’ | A(1:5); B(1:25); C (1:25) dilution in D for 3’ | A(1:5); B(1:25); C (1:25) dilution in D for 3’ /  A(1:10); B(1:50); C (1:50) dilution in D for 3’ | A(1:10); B(1:50); C (1:50) dilution in D for 3’ / A(1:20); B(1:100); C (1:100) dilution in D  for 3’ | **A(1:10); B(1:50); C (1:50) dilution in D for 3’** |

¹Novolink Polymer Detection System (Leica Microsystems, Newcastle, UK); ²Bond Polymer Refine Red Detection (Leica Microsystems, Newcastle, UK); ³ Trilogy (Cell Marque, Rocklin, CA, USA); After antigen retrieval step the reactions were wash three times in Tris solution (5mmol/L Tris; 140mM NaCl; pH 7.6) between each of the steps described.
