# Supplementary material for: Anti-mitochondrial Tryparedoxin Peroxidase Monoclonal Antibody-Based Immunohistochemistry for Diagnosis of Cutaneous Leishmaniasis
Source: Front Microbiol. 2022 Feb 28;12:790906. doi: 10.3389/fmicb.2021.790906 (PMC8918995; doi:10.3389/fmicb.2021.790906)
Supplement: Supplementary file 2 [file Table_2.pdf]

S2 Table. Details about CL positive patients

Cod	Age (years)	Gender	Municipality (Minas Gerais, Brazil)	Time of onset skin lesion	Type of lesion	Number of lesion	DE	Culture	HE	IHC-HRP	IHC-AP	PCR	Parasity load*
CL 001	3	M	Raposos	1	ulcer	1	1	1	1	1	1	1	243,45
CL 002	40	F	Água boa	1	ulcer	1	9	0	0	0	1	1	0,01
CL 003	8	M	Belo Horizonte	1	ulcer	1	1	1	1	1	1	1	30,13
CL 004	13	M	Jequitibá	1	ulcer	1	1	1	1	1	1	1	9633,19
CL 005	14	M	Santa Luzia	2	ulcer	1	1	0	1	0	0	1	3,06
CL 006	52	F	Rio Acima	2	ulcer	1	1	1	0	0	1	1	4,54
CL 007	40	M	Mariana	2	plate	1	0	1	0	0	0	1	6,87
CL 008	16	M	Santo Antônio do Rio Abaixo	2	ulcer	2	1	1	0	1	1	1	4948,81
CL 009	19	M	Santana do Riacho	2	ulcer	4	1	0	0	1	1	1	9,33
CL 010	5	F	Governador Valadares	2	ulcer	2	1	0	1	1	1	1	85,64
CL 011	70	F	Santa Luzia	2	ulcer	3	1	0	1	1	0	1	105,84
CL 012	21	M	Belo Horizonte	2	ulcer	2	1	1	0	0	1	1	239,13
CL 013	27	M	Juatuba	2	ulcer	2	1	1	1	1	1	1	64,77
CL 014	30	M	Rio Acima	2	ulcer	1	1	1	1	1	1	1	24894,54
CL 015	31	M	Belo Horizonte	2	ulcer	1	1	0	1	1	1	1	192,23
CL 016	61	F	Belo Horizonte	2	ulcer	1	0	0	0	1	0	1	0,59
CL 017	32	M	Caeté	3	ulcer	1	1	1	0	1	1	1	12675,89
CL 018	67	F	Rio Acima	3	ulcer	2	0	0	1	1	1	1	1,10
CL 019	33	M	Santa Luzia	3	ulcer	1	1	0	1	1	1	1	272,67
CL 020	36	M	Malacacheta	3	ulcer	2	9	1	0	0	1	1	1,43
CL 021	67	F	Belo Horizonte	3	ulcer and	1	1	0	1	1	1	1	206,56
CL 022	38	M	Cipotanea	3	ulcer	1	1	0	1	0	0	1	37,16
CL 023	42	M	Pedro Leopoldo	3	ulcer	2	1	1	0	1	1	1	5267,59
CL 024	42	M	Água boa	3	ulcer e plate	2	1	0	1	1	1	1	227,55
CL 025	42	M	Betim	3	ulcer and	3	1	0	0	0	0	1	0,23
CL 026	43	M	Presidente Juscelino	3	ulcer	1	1	1	1	1	1	1	41498,43
CL 027	45	M	Bebedouro	4	ulcer	2	0	0	1	1	1	1	0,04
CL 028	46	M	Ladainha	4	ulcer	15	0	0	0	1	1	1	0,55
CL 029	47	M	Sabará	4	ulcer	1	1	1	1	1	1	1	294239,53
CL 030	53	F	Sabará	4	ulcer	2	1	0	1	1	1	1	7,60

CL 031	47	M	Belo Horizonte	4	ulcer	s	1	1	1	1	1	1	218419,56
CL 032	50	M	Graminha	4	ulcer	1	1	0	1	1	1	1	0,79
CL 033	45	F	Contagem	4	ulcer	1	1	1	0	1	1	1	45,30
CL 034	50	M	Belo Horizonte	4	ulcer	1	1	1	1	0	1	1	55,63
CL 035	50	M	Sabará	4	ulcer	3	9	0	1	1	1	1	0,32
CL 036	52	M	Contagem	5	ulcer	1	1	1	0	1	1	1	4,05
CL 037	58	M	Contagem	5	ulcer	1	0	0	1	1	1	1	75,22
CL 038	62	M	Alto Caiçaras	5	ulcer e nodule	2	1	1	1	1	1	1	2245,52
CL 039	63	M	Água Boa	5	ulcer (2)	3	1	1	1	1	1	1	4908,57
CL 040	63	M	Novo Cruzeiro	6	ulcer	1	9	0	1	1	1	1	7,12
CL 041	63	M	Novo Cruzeiro	6	ulcer	1	1	1	1	1	1	1	8,75
CL 042	67	M	Tapirai	6	ulcer	5	1	1	1	1	1	1	15880,47
CL 043	56	F	Barão de cocais	7	plate	1	1	0	1	1	1	1	74,95
CL 044	69	M	Raposos	7	ulcer	3	1	1	1	1	1	1	924,51
CL 045	70	M	Ladainha	8	ulcer	2	9	1	0	0	1	1	87,55
CL 046	10	F	Belo Horizonte	8	ulcer	1	1	9	1	1	1	1	0,44
CL 047	73	M	Raposos	10	ulcer	1	1	0	0	1	1	1	2,90
CL 048	78	M	São Domingos das Dores	11	papule	3	1	0	0	1	1	1	5601,12
CL 049	84	M	São Gonçalo do Rio Preto	18	ulcer	1	1	0	1	1	0	1	45,49

Legend: (DE)Direct examination; (HE)Histopathological examination; (IHC-HRP)Immunohistochemistry with enzymes horseradish peroxidase; (IHC-FA)Immunohistochemistry with alkaline phosphatase; (qPCR)Quantitative real-time polymerase chain reaction; *expressed as the number of Leishmania parasites per µg of tissue DNA.
