# Supplementary figures and images for: Anti-mitochondrial Tryparedoxin Peroxidase Monoclonal Antibody-Based Immunohistochemistry for Diagnosis of Cutaneous Leishmaniasis
Source: Front Microbiol. 2022 Feb 28;12:790906. doi: 10.3389/fmicb.2021.790906 (PMC8918995; doi:10.3389/fmicb.2021.790906)

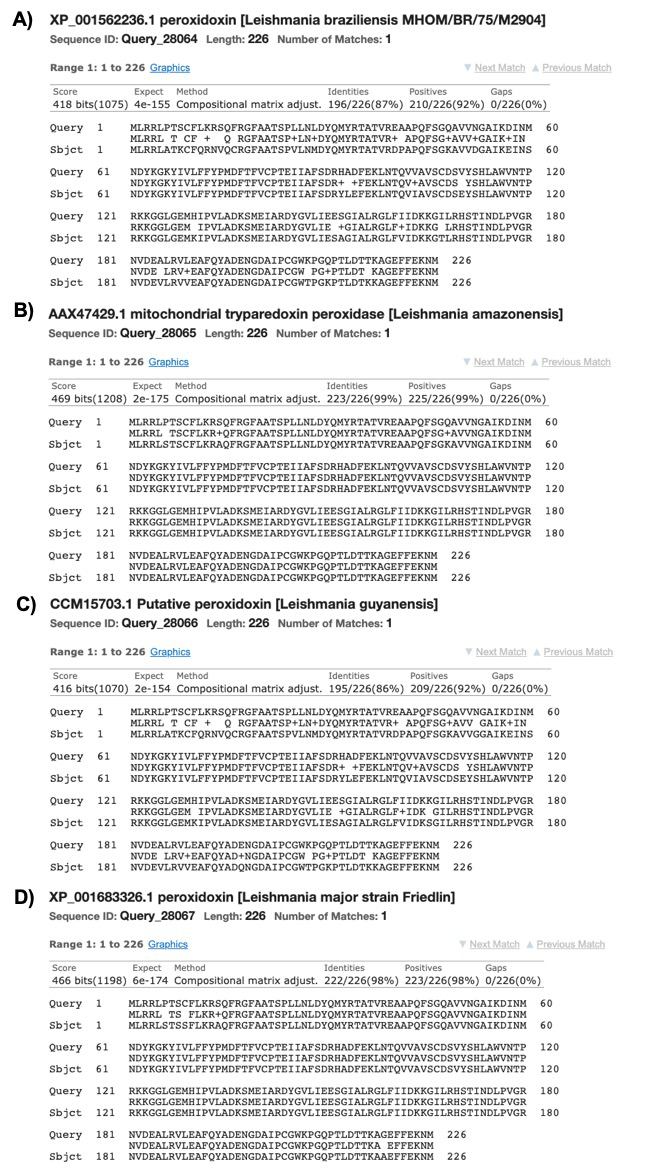

Supplement: Supplementary Figure 1 — Sequence of Leishmania infantum–mTXNPx used to produce the recombinant antigen, aligned with mTXNPx from Leishmania braziliensis (A), Leishmania amazonensis (B), Leishmania guyanensis (C), and Leishmania major (D). [file Image_1.TIF]

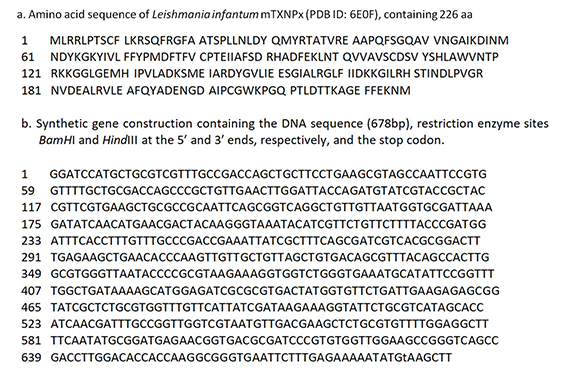

Supplement: Supplementary Figure 2 — Sequences of mTXNPx: (A) amino acid sequence of L. infantum mTXNPx (PDB ID: 6E0F) containing 226aa. (B) Synthetic gene construction containing the DNA sequence (678 bp), restriction enzyme sites BamHI and HindIII at the 5′ and 3′ ends, respectively, and the stop codon. [file Image_2.tiff]

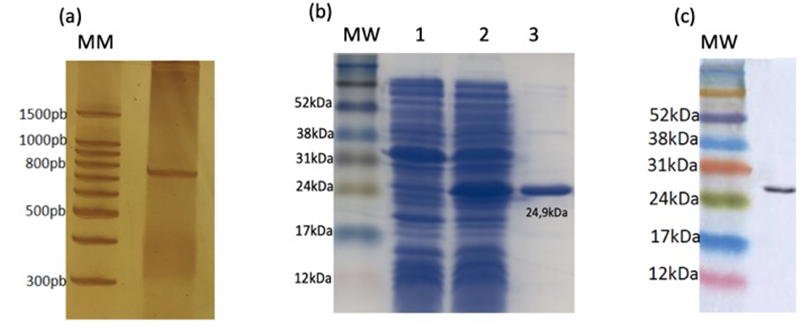

Supplement: Supplementary Figure 3 — Production of recombinant mTXNPx. (A) Polyacrylamide gel with DNA fragment of synthetic gene stained with silver, (B) 15% SDS-PAGE with lysate of culture before (1) and after (2) induction with IPTG and purified recombinant mTXNPx (3). (C) Western blotting using monoclonal 6x-His-tag antibody against recombinant mTXNPx. Molecular marker: 100 bp DNA Ladder (Promega, United States); molecular weight markers: Amersham ECL Rainbow Markers (GE Healthcare, Chicago, IL, United States). [file Image_3.TIF]

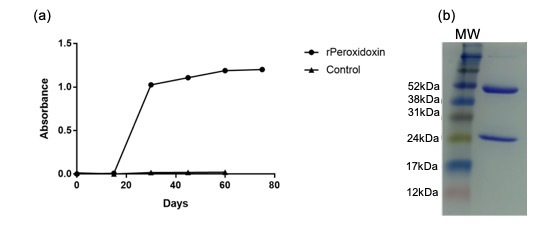

Supplement: Supplementary Figure 4 — Production of anti-mTXNPx monoclonal antibody. (A) Kinetics of antibody production (pools of sera) measured by ELISA before each immunization from mice immunized with recombinant mTXNPx or saline plus Freund’s adjuvant. (B) 15% SDS-PAGE showing purified monoclonal anti-mTXNPx antibody under denaturing conditions (fragments of ∼50 and ∼25 kDa corresponding to heavy and light chains, respectively). [file Image_4.TIF]
